# Determination of Methanol, Acetaldehyde, and Ethyl Acetate in Thousand Folds of Ethanol Sample by Headspace Gas Chromatography with Mass Spectrometry

**DOI:** 10.1155/2023/8851265

**Published:** 2023-09-16

**Authors:** Nguyen Quoc Thang, Le Van Tan

**Affiliations:** Chemical Engineering Faculty, Industrial University of Ho Chi Minh City, 12 Nguyen Van Bao, Go Vap, Ho Chi Minh City 700000, Vietnam

## Abstract

Alcohol beverages have been widely consumed in several parts of the world. In this study, volatile organic compounds in alcoholic beverages including acetaldehyde, ethyl acetate, methanol, and higher ethanol were investigated and evaluated using a headspace gas chromatograph equipped with a mass spectrometer. This study evaluated the suitability of the chromatographic system, linearity, limit of detection, and limit of quantification, accuracy, and precision of the single and simultaneous determination of acetaldehyde, ethyl acetate, and methanol in thousand folds of ethanol. Results showed that the acetaldehyde concentration in local beer samples and local manual product liqueur samples ranged from 4.65 to 13.22 mg/L and from 5.55 to 75.96 mg/L, respectively, but in local industrial product liqueur samples, acetaldehyde was not detected. Methanol was only detected in a few local beer samples and locally manually produced liqueur samples within low concentrations. Ethyl acetate was only detected in all local beer samples, but it was not present in local industrial product liqueur samples.

## 1. Introduction

Ethanol level is important to consumers for the mouthfeel and flavor of alcoholic beverages. Beer, wine, and liqueur have ethanol levels ranging from 3 to 6% (v/v), 7 to 21% (v/v), and 20 to 50% (v/v), respectively [1, 2]. This compound is presented in alcoholic beverages as a consequence of the carbohydrates' fermentation by yeasts. Volatile flavor compounds were presented in the alcoholic beverages caused by distillation procedure [2, 3]. Among these compounds, acetaldehyde is one of the naturally occurring compounds that could be found in diverse beverages (such as alcoholic drinks and juices) and foods (such as dairy products and vegetables) as well as liqueurs, wines, and brandies [4]. This compound was a flavor enhancer that was added at low concentrations to provide a pleasant fruity bouquet [5, 6]. Acetaldehyde in the most of 18 craft beer samples was from 2.02 to 19.64 mg/L and in 8 industrial beer samples was from 2.91 to 13.28 mg/L [7]. In alcoholic beverages or drinks, besides acetaldehyde, methanol is also one of the naturally occurring compounds that can be found at various levels because methanol is a byproduct that was produced due to the degradation of pectin during liqueurs' fermentation [8]. Methanol in most of the 18 craft beer samples was from 4.52 to 11.13 mg/L and in 8 industrial beer samples was not detected [7]. In the case of ethyl acetate, this is the most frequent ester in liqueurs and it is produced by the acetic bacteria metabolism and yeast during the liqueur fermentation. One of the symptoms of liqueur spoilage is a high level of ethyl acetate. It is well known that the high level of ethyl acetate in liqueur is a factor conditioning liqueur quality [9]. Ethyl acetate in most of the 18 craft beer samples was from 13.30 to 42.33 mg/L and in 8 industrial beer samples was from 13.68 to 27.79 mg/L [7].

On the other hand, ethanol, methanol, acetaldehyde, and ethyl acetate are volatile compounds whose detection and quantification in alcoholic beverages can be used as a biological indicator to identify the origin of several kinds of alcoholic drinks because the volatile compounds which characterize the beverage type are diverse and originated from raw materials. And these volatile compounds are generated during production, maturation, and storage [10].

Ethanol, acetaldehyde, methanol, and ethyl acetate in alcoholic beverages were determined by several methods. Ana Catarina et al. determined acetaldehyde, ethyl acetate, and methanol in wine spirits, brandy, and grape marc spirits by the GC-FID method [11]. Acetaldehyde and methanol in alcoholic beverages were determined by GC-MS [12], GC-FID, and GC-TCD [13, 14]. Methanol and ethanol concentrations in alcoholic drinks were detected using GC-TCD [15] and HS-GC-MS [16]. These volatile compounds were simultaneously determined by GC-FID [17]. There are such methods for the simultaneous determination of methanol, acetaldehyde, and ethyl acetate in a high level of ethanol in alcoholic drinks. This method, which is proposed in the present study, enriches the scientific literature and has a practical, applied value. Thus, the simultaneous determination of methanol, acetaldehyde, and ethyl acetate in thousand folds of ethanol in alcoholic drinks by headspace gas chromatography with mass spectrometry is a necessary method to evaluate liqueur quality.

## 2. Materials and Methods

### 2.1. Chemicals

Methanol, acetaldehyde, ethyl acetate, ethanol standard, n-butanol internal standard, acetonitrile, and water were purchased from Merck, Darmstadt, Germany. All chemical standards used for the analysis were of analytical grade. Nitrogen, hydrogen, and helium were of extrapure grade 4.5 (Air Liquide, Ho Chi Minh City, Vietnam).

Methanol, acetaldehyde, ethyl acetate, ethanol, acetonitrile standard solutions, and n-butanol internal standard solution were prepared in water solvent and stored at −18°C. These solutions were sonicated for 5 min before use.

### 2.2. Sample Preparation and Equipment

5 mL of samples was poured into 20 mL of glass headspace vials, n-butanol was added as internal standard, and then, the vials were sealed with caps lined with a silicon PTFE membrane. The vial samples were incubated for 10 min at 70°C in order to facilitate the transfer of analytes to the sample's volatile fraction. All samples were analyzed by GC-MS Thermo Fisher Scientific ISQ 72008051 gas chromatography with a mass spectrometric detector equipped split/splitless inlets, and a headspace sampling system. For separation of impurities, 1 *μ*L of the sample's volatile fraction in the headspace was injected into the GC inlet. A TG-WAXMS 30 m × 0.25 mm × 0.5 *μ*m (polyethylene glycol) capillary column was used to separate the volatile fractions under a constant flow of helium 1.2 mL/min. The duration of the analysis was 8 minutes. For increased sensitivity, other method parameters were optimized. All experimental samples were analyzed in triplicate.

### 2.3. Validation of the Method of a Single Determination of Methanol, Acetaldehyde, Ethyl Acetate, and Ethanol in Alcohol

Each of the acetaldehyde, methanol, and ethyl acetate standard solutions was prepared for calibration at 5 to 240 mg/L and 50 to 2400 mg/L of ethanol standard solution. Every single standard solution was determined in triplicate. All prepared standards were analyzed at optimized parameters by HS-GC-MS Thermo Fisher Scientific ISQ 72008051 gas chromatography. The validation method of single determination was determined by the limit of detection (LOD), the limit of quantification (LOQ), repeatability, and recovery.

The LOD is the smallest sample concentration at which the substance signal may be consistently recognized when compared to a blank run's baseline noise. For evaluating the detection limit, a signal-to-noise ratio of 3 : 1 is typically regarded adequate [18].

(1) $$\begin{array}{c} \text{LOQ}=3.3\text{ LOD.} \end{array}$$

Intra-assay and inter-assay precision levels were assessed by analyzing the quality control samples. Intra-assay was assessed using a minimum of 9 determinations at 3 concentrations/6 replicates each for the procedure. Inter-assay precision levels were evaluated in three concentrations at 5, 80, and 240 mg/L for acetaldehyde, ethyl acetate, and methanol and 50, 800, and 2400 mg/L for ethanol for six consecutive days. Intra-assay and inter-assay precision was evaluated in terms of relative standard deviation (% RSD).

Acetaldehyde, methanol, ethyl acetate, and ethanol recoveries were measured based on accuracy at 5, 80, and 240 mg/L for acetaldehyde, ethyl acetate, methanol, and 50, 800, and 2400 mg/L for ethanol.

(2) $$\begin{array}{c} \%\text{ Recovery}=\frac{\text{Actual amount}}{\text{Theoretical amount}}\times 100\text{.} \end{array}$$

### 2.4. Optimization of HS-GC-MS Parameters for the Simultaneous Determination of Methanol, Acetaldehyde, Ethyl Acetate, and Ethanol

The experiment was carried out by varying the incubation temperature of headspace at 40°C, 50°C, 60°C, 70°C, and 80°C; incubation time of the headspace at 5 min, 10 min, 15 min, 20 min, 25 min, and 30 min; inlet temperature at 150°C, 170°C, 190°C, 210°C, 230°C, and 250°C; column temperature program at 38°C and increased to 50°C with various heating rate of 3°C/min, 10°C/min, 15°C/min, and 20°C/min, to 25°C/min, where it was held for 1 min and then 50°C and increased to 170°C at 35°C/min; detector temperature at 150°C, 170°C, 190°C, 210°C, 230°C, and 250°C; carrier gas flow rate was surveyed at 1.0 mL/min, 1.2 mL/min, 1.4 mL/min, 1.6 mL/min, 1.8 mL/min, and 2.0 mL/min; split ratio at 1 : 10, 1 : 20, 1 : 40, 1 : 60, and 1 : 100. The chromatogram was recorded, and the area of the peak was calculated to choose the optimal condition.

### 2.5. Validation of the Method for Simultaneous Determination of Acetaldehyde, Methanol, Ethyl Acetate, and Ethanol through HS-GC-MS by Using Optimized Parameters

For calibration, the mixer of acetaldehyde, methanol, ethyl acetate, and ethanol standards at 5 to 240 mg/L of acetaldehyde, methanol, and ethyl acetate standard solutions and 50 to 2400 mg/L of ethanol standard solution were prepared. Every mixer standard solution was determined in triplicate. All prepared samples were analyzed at optimized parameters by HS-GC-MS Thermo Fisher Scientific ISQ 72008051 gas chromatography. The validated method simultaneous determination was determined by the selectivity factor (*α*), the limit of detection (LOD), the limit of quantification (LOQ), repeatability, and recovery.

### 2.6. Application of the Optimized Method

The optimized method was applied to 5 beer samples, 5 industrial liqueur samples, and 5 manual liqueur samples (Table 1) purchased from shops in Ho Chi Minh City, Vietnam. All samples were stored at room temperature (21°C) and protected from light. All samples were added with internal standard prior to the analysis.

## 3. Results and Discussion

### 3.1. Single Determination of Methanol, Acetaldehyde, Ethyl Acetate, and Ethanol in Alcohol by HS-GC-MS

**Figure 1:**
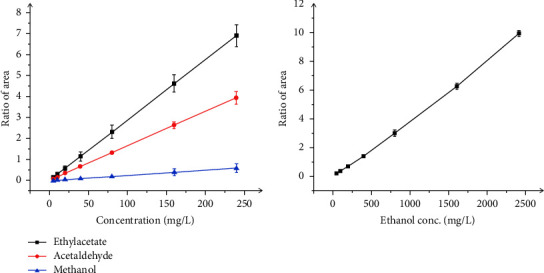
Linear regression of acetaldehyde, methanol, ethyl acetate, and ethanol.

The linearity of the headspace gas chromatography mass spectrometry method was determined at seventh concentration levels ranging from 5 to 240 mg/L of acetaldehyde, methanol, and ethyl acetate standard solutions and from 50 to 2400 mg/L of ethanol standard solution. Each of these standard solutions was incubated in the headspace at 70°C for 10 minutes, and then, the evaporative part was injected into the chromatographic system (*n* = 3). The peak area and retention time of acetaldehyde, methanol, ethyl acetate standard, and internal standard were recorded, and the mean values of the peak area ratio were plotted against the concentrations to obtain the calibration curves. Linear regression of acetaldehyde, methanol, ethyl acetate, and ethanol were *y* = (0.0164 ± 0.0008) *x* + 0.0151 ± 0.0098 (mg/L),*y* = (0.0025 ± 0.00004) *x* + 0.0008 ± 0.0003 (mg/L), *y* = (0.0298 ± 0.0064) *x* + 0.0167 ± 0.0138 (mg/L), and *y* = (0.0033 ± 0.0002) *x* + 0.0501 ± 0.0135 (mg/L), respectively (Figure 1). The good coefficients of acetaldehyde, methanol, ethyl acetate, and ethanol were 0.9998, 0.9998, 0.9999, and 0.9999, respectively.

The results of validating the determination of acetaldehyde, methanol, ethyl acetate, and ethanol are shown in Table 2. As presented in Table 2, the limit of detection (LOD) and limit of quantification (LOQ) were 0.55 mg/L and 1.83 mg/L for ethanol, 0.63 mg/L and 2.09 mg/L for methanol, 0.52 mg/L and 1.72 mg/L for acetaldehyde, and 0.51 mg/L and 1.70 mg/L for ethyl acetate, respectively. The LOD in this research was better than that in other documents. Helena et al. presented a LOD of 0.85 mg/L for acetaldehyde and 0.75 mg/L for acetone, ethanol, and methanol [18]. Pontes et al. showed that the LOD was 0.87 mg/L for methanol, 0.51 mg/L for acetaldehyde, and 0.82 mg/L for ethyl acetate [19]. The method in this study has good accuracy, precision, linearity, and efficiency for the quantification of acetaldehyde, methanol, ethyl acetate, and ethanol.

### 3.2. Simultaneous Determination of Methanol, Acetaldehyde, Ethyl Acetate, and Ethanol in Alcohol by HS-GC-MS

#### 3.2.1. The Optimized Parameters

In HS-GC-MS, the samples must be held in the headspace system at high temperatures and a reproducible equilibrium must be established between a solution sample and a headspace. The chromatographic process depends on the analytical conditions including detector temperature, inlet temperature, column temperature program, flow rate, split ratio, headspace temperature, and incubation time of the headspace. Therefore, the study of headspace conditions and GC-MS parameters in the multicomponent analysis is necessary for the HS-GC-MS method.

#### 3.2.2. Optimization of Incubation Temperature of Headspace

**Figure 2:**
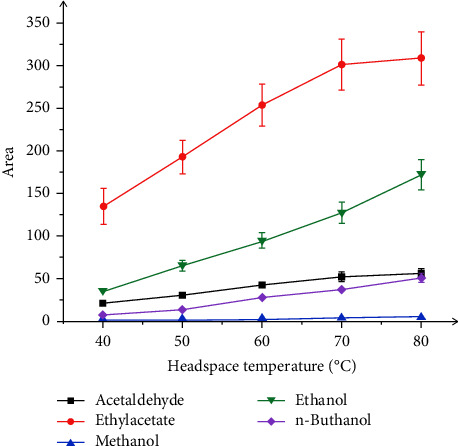
Effect of headspace temperature on the peak area of ethanol, methanol, ethyl acetate, and acetaldehyde.

In the headspace technique, the sample was put into a sealed vial which was heated to an essential temperature for a period of time. More volatile compounds will tend to move into the headspace above the solution sample. The incubation temperature of headspace was one of the factors affecting the volatile ability of compounds. In this study, after some trials of different headspace temperatures for 10 min, 80°C was selected as a headspace temperature because of the great peak surface. The results are shown in Figure 2.

#### 3.2.3. Optimization of Incubation Time of the Headspace

**Figure 3:**
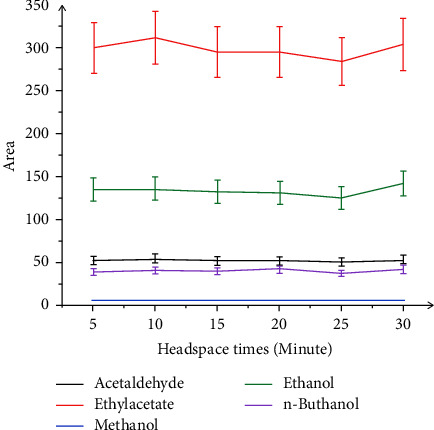
Effect of incubation time of the headspace on the peak area of ethanol, methanol, ethyl acetate, and acetaldehyde.

Solvent-vapor equilibria in the headspace play an important role in the headspace analysis. The more volatile compounds can be evaporated into the headspace, and more volatile compounds will be injected into the column of the GC. If the sample is incubated for a too short time, less of the volatile compounds will be in the headspace, which can affect the overall peak area. In this study, the incubation time of the headspace was in the range of 5–30 minutes. The results shown in Figure 3 show that those equilibration time periods longer than 10 min do not yield a significant increase in the peak area. In the case of ethanol, methanol, ethyl acetate, and acetaldehyde, the incubation time longer than 20 minutes shows a high peak area. It is of interest to note that the incubation time of the samples was 10 minutes.

#### 3.2.4. Optimization of Inlet Temperature

**Figure 4:**
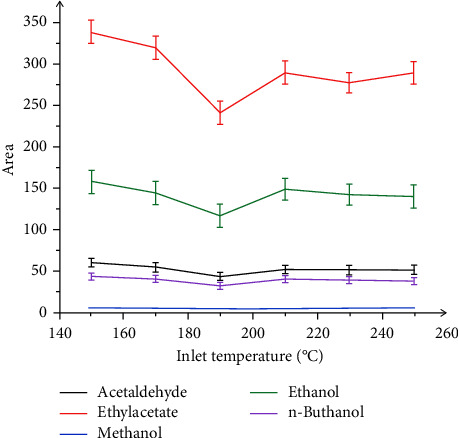
Effect of inlet temperature on the peak area of ethanol, methanol, ethyl acetate, and acetaldehyde.

The inlet was to set an optimal temperature which helped the analytes quickly vaporize. It was not only too high to cause the breakdown of these compounds but also not too low to decrease the sensitivity due to the compounds which did not vaporize. Inlet temperature was not adjusted higher or lower to optimize the performance in each different analysis. In our study, the inlet temperature was set in the range from 150 to 250°C. The results are presented in Figure 4. Figure 4 presents that the peak area of ethanol, methanol, ethyl acetate, and acetaldehyde in the chromatogram decreased with increasing temperature in the inlet. The optimal inlet temperature was set at 150°C.

#### 3.2.5. Optimization of GC Oven Temperature

**Figure 5:**
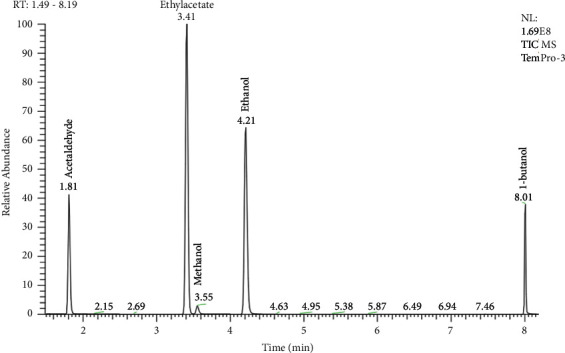
Chromatograms of ethanol, methanol, ethyl acetate, and acetaldehyde in the GC oven temperature set from 38°C and increased to 50°C at 3°C/min, where it was held for 1 min and then 50°C and increased to 170°C at 35°C/min. The retention time periods *t*_*R*_ of acetaldehyde, ethyl acetate, methanol, ethanol, and butanol were 1.81 min, 3.41 min, 3.55 min, 4.24 min, and 8.01 min, respectively.

The programmed temperature was related to the analysis which was performed on the instrument. For acetaldehyde, ethyl acetate, methanol, and ethanol, the program can be operated at an inlet temperature of 150°C, with the column program starting from 38°C and increased to 50°C at several heating rates from 3°C/min to 25°C/min, where it was held for 1 min and then from 50°C and increased to 170°C at 35°C/min. In these chromatograms, the peaks of ethyl acetate and methanol overlapped at heating rates of 10, 15, 20, and 25°C/min. Otherwise, the peak of acetaldehyde, ethyl acetate, methanol, and ethanol can be separated at a heating rate of 3°C/min. So, the optimization of temperature for the column program to operate was an inlet temperature of 150°C with column program from 38°C and increased to 50°C at 3°C/min, where it was held for 1 min and then 50°C and increased to 170°C at 35°C/min. This temperature column program was set for the simultaneous determination of methanol, acetaldehyde, ethyl acetate, and ethanol in alcohol. The chromatogram of this temperature column program is shown in Figure 5.

Table 3 shows the chromatographic system data of simultaneous analysis of acetaldehyde, ethyl acetate, methanol, ethanol, and butanol at heating rates of 3, 5, 10, 15, 20, and 25°C/min. The results showed that when the heating rate increased, the retention time of the analytes decreased. It was because gas chromatography relies on the evaporation temperature of the analytes to separate the analytes contained in the sample background. Therefore, when the temperature increases rapidly, the analytes will be eluted out of the column faster, which causes the peak of analytes, which takes similar retention time to overlap each other. It is easy to completely overlap the low-intensity peaks. So, the chromatographic spectrum will only show high-intensity peaks.

#### 3.2.6. Optimization of Detector Temperature

**Figure 6:**
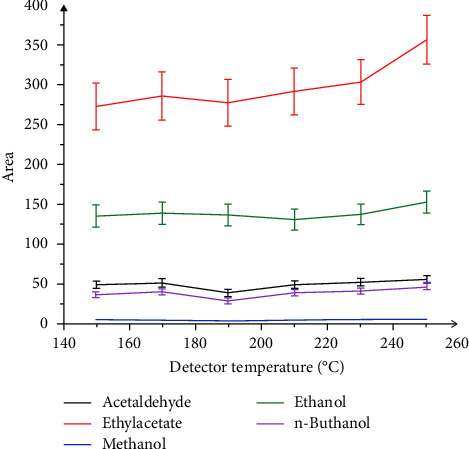
Effect of detector temperature on peak areas of ethanol, methanol, ethyl acetate, and acetaldehyde.

After the volatile compounds moved the length of the GC column, they were entered into the mass spectrometer and were fragmented into an ion by an electron ionization technique. In the electron ionization technique, an electron that was produced by a filament was accelerated and knocks an electron out of the molecule to produce a molecular ion. These molecular ions were detected by an electron multiplier, which essentially turned the ionized mass fragment into an electrical signal that was then detected. In this study, the detector temperature was set at 150, 170, 190, 210, 230, and 250°C. The effect of the detector temperature on the peak areas of ethanol, methanol, ethyl acetate, and acetaldehyde is shown in Figure 6. The results showed that at 250°C, the peak area of ethanol, methanol, ethyl acetate, and acetaldehyde was the highest. The optimal detector temperature was set at 250°C.

#### 3.2.7. Optimization of Mobile Phase Flow Rate

The mobile phase flow rate changed from 1.0 to 2.0 mL/min. The results showed that the flow rate increased and the retention time of the analytes decreased. It was due to the fact that the carrier gas plays the role of the mobile phase eluting the analytes out of the column, the analytes move out of the column as soon as possible. The change in the mobile phase flow rate causes the change of peak areas of ethanol, methanol, ethyl acetate, and acetaldehyde (Table 3). This may be caused by the decrease in ionization efficiency due to the nonstoichiometric ratio of air and the dilution of gas eluted from the column. Thus, the mobile phase flow rate of 1.2 mL/min was selected as the optimal value (Table 4).

#### 3.2.8. Optimization of Slip Ratio

**Figure 7:**
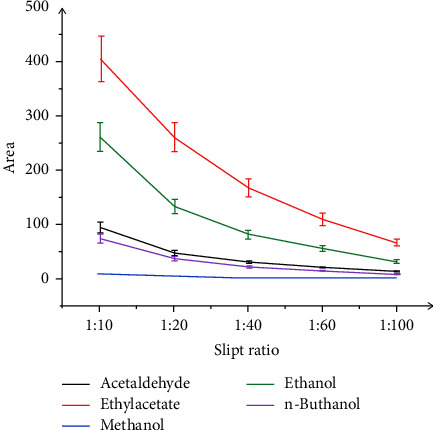
Effect of split ratio on the peak area of ethanol, methanol, ethyl acetate, and acetaldehyde.

One of the substantial parameters affecting method sensitivity was the split ratio. Increasing the split ratio leads to a decrease in the amounts of analytes introduced into the column. Therefore, the splitless injection technique will be the highest sensitivity. In the case of the headspace technique, ethanol, methanol, ethyl acetate, and acetaldehyde were evaporated into the headspace upper of the solution, so that the impurity compounds could be removed. In an alcohol sample, the ethanol amount was steadily higher than other compounds. Injection in the splitless mode or at low split ratios may potentially lead to problems with the methanol peak which can be overlapped by a large ethanol peak, analyte peak shape, and efficiency of resolution. Split ratios were studied at 1 : 10, 1 : 20, 1 : 40, 1 : 60, and 1 : 100.  Figure 7 shows the effect of the split ratio on the peak area of ethanol, methanol, ethyl acetate, and acetaldehyde. At a split ratio 1 : 10, the peak area of these analytical compounds was large and this ratio was set at the optimal parameter.

#### 3.2.9. System Suitability

The mixture of ethanol, methanol, ethyl acetate, and acetaldehyde was injected into HS-GC-MS in six replications under the optimal conditions mentioned above. The peak width, retention time, resolution, symmetry, and theoretical plates were recorded to assess the suitability of the analytical instrumentation conditions. The results in Table 5 show the theoretical plates >2000, resolution >1.5, symmetry in the range 0.9–1.1, and the relative standard deviation of retention time <2%. These system suitability parameters were obtained as acceptance criteria from the International Conference on Harmonization (ICH) [20, 21]. The method was suitable for the simultaneous analysis of ethanol, methanol, acetaldehyde, and ethyl acetate.

#### 3.2.10. Optimized Parameters

Headspace injector conditions include the following:
  - Incubation temperature of headspace: 80°C
  - The incubation time of the headspace: 10 min
Chromatographic conditions include the following:
  - Column: TG-WAXMS 30 m × 0.25 mm × 0.5 *μ*m (polyethylene glycol)
  - Detector temperature: 250°C
  - Inlet temperature: 150°C
  - GC oven temperature: 38°C and increased to 50°C at 3°C/min, where it was held for 1 min and then 50°C and increased to 170°C at 35°C/min
  - Injector volume: 400 *μ*L
  - Slipt ratio: 1 : 10
  - Mobile phase flow rate: 1.2 mL/min

### 3.3. Validation of the Method of Simultaneous Determination of Methanol, Acetaldehyde, Ethyl Acetate, and Ethanol in Alcohol by HS-GC-MS

#### 3.3.1. Linear Regression

The linearity of simultaneous determination of methanol, acetaldehyde, ethyl acetate, and ethanol in the headspace gas chromatography mass spectrometry method was determined at seven concentration levels ranging from 5 to 240 mg/L of acetaldehyde, methanol, and ethyl acetate standard solutions and from 50 to 2400 mg/L of ethanol standard solution. Each of these standard solutions was incubated in the headspace at 70°C for 10 minutes, and then, the evaporative part was injected into the chromatographic system (*n* = 3). Recording the peak area of acetaldehyde, methanol, ethyl acetate, ethanol standard, and n-butanol internal standard, the mean values of the peak area ratio was plotted against the concentrations to obtain the calibration curves. Linear regression and good coefficients of acetaldehyde, methanol, ethyl acetate, and ethanol are shown in Table 6.

Table 5 shows that the concentration and peak area of acetaldehyde, ethyl acetate, and methanol in the concentration range from 5 to 240 mg/L and ethanol in the concentration range from 50 to 2400 mg/L correlated with linear relationship *r*^2^ > 0.999.

#### 3.3.2. Limit of Detection and Limit of Quantification

The limit of detection and limit of quantification of the method are presented in Table 7. Table 7 presents the results obtained from the simultaneous determination of acetaldehyde, methanol, ethyl acetate, and ethanol in alcohol samples using the proposed method.

#### 3.3.3. Repeatability

Intra-assay and inter-assay precision levels were assessed by analyzing the quality control samples. Intra-assay was assessed using a minimum of 9 determinations at 3 concentrations/6 replicates each for the procedure. Inter-assay precision levels were evaluated in three concentrations at 5, 80, and 240 mg/L for acetaldehyde, ethyl acetate, and methanol and 50, 800, and 2400 mg/L for ethanol for six consecutive days. Intra-assay and inter-assay precisions were evaluated in terms of relative standard deviation (% RSD). The RSD of intra-assay and inter-assay precisions in Table 8 was less than 4% at three concentrations of acetaldehyde, methanol, ethyl acetate, and ethanol.

#### 3.3.4. Recovery

Acetaldehyde, methanol, ethyl acetate, and ethanol recovery were measured based on accuracy at 5, 80, and 240 mg/L for acetaldehyde, ethyl acetate, and methanol and 50, 800, and 2400 mg/L for ethanol. Recovery of acetaldehyde, methanol, ethyl acetate, and ethanol was found to be 95–102%, 97–103%, 98–105%, and 99–107%, respectively (Table 9).

The results showed that the limit of detection, the limit of quantification, linear range, repeatability, and recovery efficiency of the simultaneous method were good, meeting the requirements for method validation of AOAC by GC-MS equipment.

#### 3.3.5. Advantages and Disadvantages of Simultaneous Determination of Methanol, Acetaldehyde, Ethyl Acetate, and Ethanol in Alcohol by HS-GC-MS

- Saving analysis time: By only one injection, the simultaneous concentration of methanol, acetaldehyde, ethyl acetate, and ethanol could be determined in samples. The total time required for the chromatographic analysis was 9 min. Several authors have developed GC methods for the simultaneous determination of methanol, acetaldehyde, ethyl acetate, and ethanol. One comparable study was conducted by Schlatter et al. who developed and validated a method for the simultaneous determination of acetaldehyde, methanol, acetone, and ethanol. The total time required for the chromatographic analysis was 15 min, which was 1.5 fold higher than our method [22].
- Cost savings: By only a single injection, the concentration of methanol, acetaldehyde, ethyl acetate, and ethanol in the samples can be determined simultaneously, so the cost of the analysis is smaller than in the case of individual substances.
- Minimizing errors in the analysis process: In the simultaneous analysis process, the steps of the analysis were minimized compared to the single analysis process, thereby limiting the errors arising from the manipulation process.
- The analytes in the sample have different concentrations. So, it is difficult to process the samples, especially with trace concentration. To minimize this drawback, a standard addition method was performed to decrease the difference in concentration between the analytes.

#### 3.3.6. Application of Simultaneous Determination of Methanol, Acetaldehyde, Ethyl Acetate, and Ethanol in Alcohol by HS-GC-MS

Because the ethanol concentration in the alcohol samples was at 5–40%, the samples should be diluted to analyze at the appropriate ratio. Meanwhile, methanol, acetaldehyde, and ethyl acetate concentrations were lower than ethanol concentrations. Therefore, the dilution samples have done a standard addition at 5 mg/L for each methanol, acetaldehyde, and ethyl acetate, and an internal standard was added into the dilution sample.  Table 10 presents the actual sample analysis results.

Ethanol concentration in liqueur product samples varies for each product group. Ethanol concentration in beer was in a range from 4.5% to 5.9%; meanwhile, the ethanol concentration in local liqueur samples (industrial products and manual products) was in a range from 25.7% to 39.6%. These results were in accordance with the declaration of the manufacturers. Most of the beer samples did not contain methanol or contained a little methanol level. In contrast, the content of ethyl acetate in beer samples was high, in the range of 30–40 mg/L. Meanwhile, the content of acetaldehyde ranged from 4.6 to 13.2 mg/L. Beer was produced through fermentation and then filtration without distillation. So, beer samples have a lot of impurities in them, such as acetaldehyde and ethyl acetate.

Industrial liqueur products have no or contain very little methanol, acetaldehyde, and ethyl acetate (except for RNUL products which contain above 40 mg/L of acetaldehyde and ethyl acetate). Most industrial liqueur products were produced using multistage distillation towers to distillate and help separate and decrease impurities in the liqueur. In the group of manual liqueur products, the ethanol concentration is from 25.6 to 37.8%, which depends on the needs of each product. Most of the manual liqueur product samples contained acetaldehyde, but its significant difference lies in the levels of different production facilities. In contrast, ethyl acetate and methanol concentrations did not present or were found very little in manual liqueur products. The reason is that all local artisanal distilleries use simple or self-designed distillation equipment. So, the process of separating ethanol from impurities does not completely depend on the system and conditions of the distillation process. Therefore, the results in the group of manual liqueur products are significantly different in impurity concentration between production facilities. The methanol, acetaldehyde, and ethyl acetate concentrations in these liqueur products were less than these concentrations in several documented previous studies. According to Kokkinakis et al., the concentrations of acetaldehyde, ethyl acetate, and methanol in bottled spirits and in-bulk spirits were 297.58 mg/L, 429.16 mg/L, and 698.02 mg/L and 199.75 mg/L, 1067.66 mg/L, and 781.20 mg/L, respectively [23]. The methanol and acetaldehyde contents of fermented plant beverages in Thailand were less than 29 mg/L and 45 mg/L, respectively [24].

## 4. Conclusions

This study was taken to analyze the simultaneous concentration of acetaldehyde, methanol, ethyl acetate, and ethanol in alcohol, which are most commonly consumed by the habitants of Ho Chi Minh City, Vietnam. The HS-GC-MS parameters for simultaneous determination of these volatile compounds were optimized. The simultaneous method was validated. In beer samples, there were impurities such as acetaldehyde and ethyl acetate because the production process did not go through distillation. In industrial liqueur products, there is very little methanol, acetaldehyde, and ethyl acetate because multistage distillation towers were used to distillate to help separate and decrease impurities in the liqueur. For manual liqueur products, there are still impurities such as methanol and acetaldehyde because simple or self-designed distillation equipment was used to separate impurities during the production process.

## Figures and Tables

**Table 1:** Sample name, number of samples, and number of individuals for each sample.

No.	Sample	Production year	Alcohol content of manufacturer's data (%, v/v)	No. of the analysis sample	No. of individuals per sample
*Beer samples*					
1	BBV	2022	5	20	30
2	BHD	2022	5	20	30
3	BLR	2022	5	20	30
4	BSG	2022	5	20	30
5	BTG	2022	5	20	30

*Industrial liqueur samples*					
6	VKNH	2022	30	20	30
7	RDBT	2022	30	20	30
8	RNUL	2022	40	20	30
9	VKM	2022	30	20	30
10	VKG	2022	30	20	30

*Manual liqueur samples*					
11	TCHĐ1	2022	40	20	30
12	TCHĐ2	2022	25	20	30
13	TCHH1	2022	30	20	30
14	TCHH2	2022	30	20	30
15	TCHV	2022	30	20	30

**Table 2:** The results of validating the determination of acetaldehyde, methanol, ethyl acetate, and ethanol.

The validated values	Ethanol	Methanol	Acetaldehyde	Ethyl acetate
Linear range (mg/L)	5–2500	5–240	5–240	5–240

Linear relationship (*r*^2^)	0.9999	0.9998	0.9998	0.9999

Limit of detection (mg/L)	0.55	0.63	0.52	0.51

Limit of quantification (mg/L)	1.83	2.09	1.72	1.70

*Relative standard deviation (%RSD) at*:				
(1) Low concentration	50 mg/L: 2.11	5 mg/L: 2.53	5 mg/L: 2.78	5 mg/L: 2.15
(2) Middle concentration	800 mg/L: 0.98	80 mg/L: 1.43	80 mg/L: 2.13	80 mg/L: 2.38
(3) High concentration	2400 mg/L: 0.42	240 mg/L: 1.70	240 mg/L: 2.93	240 mg/L: 2.34

*Reproducibility (%RSD) at*:				
(1) Low concentration	50 mg/L: 3.56	5 mg/L: 1.15	5 mg/L: 2.42	5 mg/L: 2.78
(2) Middle concentration	800 mg/L: 1.31	80 mg/L: 1.74	80 mg/L: 2.68	80 mg/L: 2.75
(3) High concentration	2400 mg/L: 1.00	240 mg/L: 1.78	240 mg/L: 1.32	240 mg/L: 2.94

*Assay recovery (%H) at*:				
(1) Low concentration	50 mg/L: 94.14–104.11	5 mg/L: 96.28–103.25	5 mg/L: 95.89–106.15	5 mg/L: 97.68–101.95
(2) Middle concentration	800 mg/L: 98.42–101.43	40 mg/L: 98.94–103.88	80 mg/L: 95.49–103.35	80 mg/L: 98.8–104.72
(3) High concentration	2400 mg/L: 98.76–101.64	240 mg/L: 97.42–102.00	240 mg/L: 97.38–101.13	240 mg/L: 97.87–101.94

**Table 3:** Chromatographic system data: retention times, selectivity factor, peak area, resolution, symmetry, and theoretical plates at several heating rates.

Compounds	Heating rate (°C/min)	Retention time *t*_*R*_ (min)	Peak area	Symmetry	Theoretical plates (*N*)	Selectivity factor (*α*)	Resolution (Rs)
Acetaldehyde	3	1.81	132912975	1.08	14560	4.14	26.67
	5	1.81	141684515	1.00	20967	3.90	26.91
	10	1.79	131957565	1.17	14240	3.78	19.43
	15	1.78	148895708	1.08	10346	3.21	17.07
	20	1.78	143766294	1.00	14082	2.81	17.57
	25	1.76	150403601	1.17	10115	2.34	12.67

Ethyl acetate	3	3.41	401766478	1.00	51680	1.07	2.55
	5	3.29	401137946	1.17	48107	1.36	12.00
	10	3.15	364177594	0.92	24806	1.30	7.86
	15	3.06	412759798	1.22	23409	1.25	6.71
	20	3.01	383032531	0.96	22650	1.22	6.00
	25	2.71	426593713	1.22	18360	1.18	4.29

Methanol	3	3.55	290731692	1.13	80656	1.29	11.00

Ethanol	3	4.21	290731692	0.96	57875	2.31	54.29
	5	4.01	210622985	0.85	71467	1.96	43.33
	10	3.70	169468067	1.08	60844	1.78	33.82
	15	3.53	187747057	0.92	55382	1.72	30.36
	20	3.43	142977205	0.92	52288	1.69	29.09
	25	3.01	135532318	0.92	40267	1.74	26.36

Butanol	3	8.01	89132249	0.92	209502	—	—
	5	6.61	87923903	0.82	194187	—	—
	10	5.56	20142136	0.82	197847	—	—
	15	5.20	22625365	1.00	173059	—	—
	20	5.03	22851934	0.82	161926	—	—
	25	4.46	22713765	1.22	127306	—	—

**Table 4:** Chromatographic system data: retention times, selectivity factor, peak area, resolution, symmetry, and theoretical plates at several mobile phase flow rates.

Compounds	Mobile phase flow rate (mL/min)	Retention time *t*_*R*_ (min)	Peak area	Symmetry	Theoretical plates (*N*)	Selectivity factor (*α*)	Resolution (Rs)
Acetaldehyde	1.0	1.98	132912975	1.08	9801	4.23	24.43
	1.2	1.81	263343533	1.06	10625	4.14	21.33
	1.4	1.68	231957565	1.04	12544	4.38	21.71
	1.6	1.58	248895708	1.08	11095	4.25	22.00

Ethyl acetate	1.0	3.69	40166478	0.96	60516	1.07	2.00
	1.2	3.41	562827701	1.02	29070	1.07	10.15
	1.4	3.20	574141983	1.08	25600	1.38	54.29
	1.6	3.01	588579335	1.04	29584	1.39	9.73
	1.8	2.86	575987017	1.02	20449	1.39	9.33
	2.0	2.73	549110283	1.08	18632	1.39	9.07

Methanol	1.0	3.84	20731692	1.04	94372	1.29	11.00
	1.2	3.55	43102960	1.00	56011	1.29	10.15

Ethanol	1.0	4.54	290731692	1.08	51529	2.17	51.86
	1.2	4.21	746495376	1.04	57875	2.31	54.29
	1.4	3.95	484352051	1.13	39006	2.44	55.86
	1.6	3.74	451993075	1.00	34969	2.53	53.20
	1.8	3.56	485375812	0.96	41383	2.62	62.46
	2.0	3.41	312404571	0.98	37969	2.70	63.38

Butanol	1.0	8.17	89132249	1.13	296661	—	—
	1.2	8.01	205425091	1.04	209502	—	—
	1.4	7.86	205427572	1.01	274576	—	—
	1.6	7.73	204673487	1.04	195111	—	—
	1.8	7.62	212624219	1.06	258064	—	—
	2.0	7.53	20666047	0.95	252004	—	—

**Table 5:** System suitability.

		Peak width	Retention time *t*_*R*_ (min)	Resolution (Rs)	Symmetry	Theoretical plates (*N*)
Acetaldehyde	1	0.08	1.82	17.71	0.92	8252
	2	0.09	1.82	15.94	0.90	6522
	3	0.08	1.81	17.82	0.89	8162
	4	0.09	1.82	15.94	0.94	6521
	5	0.09	1.82	15.94	0.96	6521
	6	0.08	1.81	17.78	0.97	8190

Ethyl acetate	1	0.10	3.41	1.61	1.00	18613
	2	0.11	3.41	1.53	1.02	15386
	3	0.10	3.41	1.71	1.04	18613
	4	0.11	3.41	1.53	1.06	15384
	5	0.11	3.41	1.53	1.04	15384
	6	0.10	3.41	1.65	1.03	18605

Methanol	1	0.08	3.56	5.96	1.08	31614
	2	0.08	3.56	6.55	1.11	31621
	3	0.07	3.56	6.56	1.21	41287
	4	0.08	3.56	5.96	1.06	31613
	5	0.08	3.56	5.70	1.04	31613
	6	0.07	3.55	6.62	1.07	41151

Ethanol	1	0.14	4.21	37.98	1.07	14479
	2	0.12	4.21	39.98	1.07	19709
	3	0.13	4.21	39.98	1.04	16792
	4	0.14	4.21	37.98	1.03	14479
	5	0.15	4.21	34.53	1.09	12613
	6	0.13	4.21	39.98	1.13	16793

**Table 6:** Linear regression of acetaldehyde, ethyl acetate, methanol, and ethanol.

Compound	Concentration range (mg/L)	Linear regression	Linear relationship (*r*^2^)
Acetaldehyde	5–240	*y* = (0.01360 ± 0.00093)*x* + (0.00501 ± 0.00036)	0.9999
Ethyl acetate	5–240	*y* = (0.0790 ± 0.00568)*x* − (0.02641 ± 0.00053)	0.9998
Methanol	5–240	*y* = (0.00125 ± 0.00006)*x* − (0.00043 ± 0.00011)	0.9998
Ethanol	50–2400	*y* = (0.00363 ± 0.00015)*x* − (0.01855 ± 0.00053)	0.9999

**Table 7:** Limit of detection and limit of quantification of the HS-GC-MS method.

	Acetaldehyde	Ethyl acetate	Methanol	Ethanol
Limit of detection (mg/L)	0.52	0.51	0.72	0.61
Limit of quantification (mg/L)	1.74	1.72	2.41	2.03

**Table 8:** The repeatability of the HS-GC-MS method.

Compound	Acetaldehyde			Ethyl acetate			Methanol			Ethanol		
Concentration (mg/L)	5	80	240	5	80	240	5	80	240	50	800	2400

*Intra-day precision of the method*												
RSD (%)	1.88	1.77	1.00	1.45	2.39	0.88	2.96	1.77	0.64	2.37	1.69	0.64

*Inter-day precision of the method*												
RSD (%)	2.24	1.29	0.72	2.60	1.23	0.46	1.28	2.82	0.96	1.20	1.94	1.30

**Table 9:** Recovery of the HS-GC-MS method.

Concentration level	% recovery			
	Acetaldehyde	Ethyl acetate	Methanol	Ethanol
Low concentration	94.73–101.39	96.65–102.94	100.7–104.21	103.79–106.79
Middle concentration	98.45–101.36	99.02–102.31	97.85–104.68	99.19–103.63
High concentration	99.91–101.96	98.97–100.05	99.69–102.40	99.19–102.96

**Table 10:** Concentrations of methanol, acetaldehyde, ethyl acetate, and ethanol in alcohol samples in Ho Chi Minh City.

No		Samples	Acetaldehyde (mg/L)	Ethyl acetate (mg/L)	Methanol (mg/L)	Ethanol (%)
1	Local beer samples	BBV	9.88	33.53	0.63	4.53
2		BHD	4.65	41.36	n/d	4.80
3		BLR	13.22	27.53	1.43	4.51
4		BSG	7.59	8.83	n/d	5.86
5		BTG	6.89	35.41	n/d	5.46

6	Local liqueur samples (industrial products)	VKNH	n/d	n/d	n/d	33.07
7		RDBT	n/d	n/d	n/d	33.24
8		RNUL	46.14	40.03	n/d	39.60
9		VKM	n/d	n/d	n/d	29.96
10		VKG	n/d	n/d	n/d	29.57

11	Local liqueur samples (manual products)	TCHD1	39.80	n/d	1.77	37.77
12		TCHD2	6.95	n/d	n/d	25.68
13		TCHH1	5.55	4.57	n/d	32.41
14		TCHH2	8.83	32.00	3.69	29.21
15		TCHV	75.96	n/d	68.75	32.75

*Note.* n/d: not detected (below detection limit).

## Data Availability

The data that support the findings of this study are available from the corresponding author upon reasonable request.
